# Association between olfactory function and inhibition of emotional competing distractors in major depressive disorder

**DOI:** 10.1038/s41598-020-63416-7

**Published:** 2020-04-14

**Authors:** Fang Wang, Jin Jin, Jun Wang, Ruoqiao He, Kaiyun Li, Xiaonan Hu, Yongchao Li, Yuncheng Zhu

**Affiliations:** 1 Shanghai Yangpu Mental Health Center, Shanghai, 200093 China; 2 Shanghai Mental Health Center, Shanghai Jiao Tong University School of Medicine, Shanghai, 200030 China; 3 Silver School of Social Work, New York University, New York, 10003 USA; 4 University of Jinan, Jinan, 250022 China

**Keywords:** Olfactory system, Depression

## Abstract

We aimed to investigate the changes of olfaction of major depressive disorder (MDD) before and after medical treatment, and to preliminarily scrutinize the association between the olfactory function and the severity of depressive symptoms, response inhibition, and emotional responding. Forty-eight medicine-naïve MDD patients plus 33 healthy controls (HC) matched on gender, ages, and level of education, were recruited in the test group. The Chinese Smell Identification Test (CSIT), Self-reported Olfactory Scale (SROS), 17-item Hamilton Depression Rating Scale (HAMD-17), Hamilton Anxiety Rating Scale (HAMA), and mean reaction time/accuracy rate (ΔMRT) of emotional Stroop test were measured. The patients were assessed before the treatment (baseline) and 3 months after the treatment (follow-up). The data at the baseline level were measured then associated using multiple linear regression stepwise analysis. The MDD patients had lower scores of the CSIT and SROS and longer ΔMRT at baseline level compared to HC while the ΔMRT of MDD patients remained longer after 3-month treatment (p’s < 0.05). At the baseline level, the regression equation including age and ΔMRT of negative word-color congruent (NEG-C), was finally observed as follows: y(CSIT) = 10.676–0.063 × 1–0.002 × 2, [x_1_ = the age(y), x_2_ = the NEG-C (ms)]. The olfactory function of MDD appears to be correlated negatively with the age and the ΔMRT of negative stimuli before treatment. After the remission of MDD, the olfactory dysfunction was improved, which might be regarded as a responding phenotype of brain function of MDD rather than the emotional responding.

## Introduction

Major depressive disorder (MDD) and olfaction disorder (OD) are both chronic diseases that affect human being’s life quality^1^. A systematic review on ten studies chosen for using the Sniffin’ Sticks Test and the 40-item Smell Identification Test assessed to reveal the relationship between depression and OD. Results show that both of the olfactory threshold, olfactory discrimination, and olfactory identification were influenced by depression^2^. Furthermore, symptoms of depression worsen with the severity of olfactory loss^3^. It was demonstrated that olfactory bulbectomy, as a well-known method, could induce an animal model of depression in animal experiment^4^. In depression-related brain areas including hippocampus, frontal cortex and hypothalamus, the turnover of serotonin and dopamine decreases in the olfactory bulbectomized rats^5^. Similarly, in human studies, Cory *et al*.^6^ found that patients with depression have decreased activities in the left medial orbitofrontal cortex (OFC) accompanied with the defect of the olfactory identification. However, Zucco *et al*.^7^ argued that the changes mentioned above occur in patients with the MDD only, not with the mild or moderate depressive disorder. In the neural circuits involved in emotional processing^8^, these key structures such as amygdala, hippocampus, anterior cingulate cortex(ACC) and OFC, are the imperative functional regions of the human olfactory map^9,10^.

Emotional response originates from the hypothalamus, whereas the activated component is located at the amygdala. Furthermore, through an evolutionary view on the neural circuits, the amygdaloid complex has widespread connections with subcortical structures, this extended amygdala takes a position to the allocortex (olfactory cortex and hippocampus)^8,11^. Based on these overlapping neural networks, it is highly doubted that the depressed patients may have olfactory dysfunction correlated with emotional dysregulation.

In addition, the correlation between cognitive dysfunction and OD may be related to the mutual pathways of OFC and subcortical structures^12,13^. The OFC and rostral insula bilaterally^14^ are the secondary olfactory neuroanatomic structures taking charge of chemosensory processing^15^ while the primary olfactory structures generate from the piriform cortex, entorhinal cortex, amygdala, and hippocampus^16^ receiving the inputs from the tractus olfactoriusta projected from the olfactory bulb. Therefore, the olfactory system and the emotion have many common structures, which provide a framework for bridging gaps between the cognitive function and the olfactory function via the similar neuropathological basis of depression^17^.

In the neuropsychology, the emotional Stroop effect is commonly used to evaluate the response inhibition with emotional responding in the field of cognitive function^18^. Under the overlapping controls of the OFC^19^, there are reasons to believe that MDD may have correlated dysfunction between cognition and olfaction. The above background shed the light on the purpose of the research to evaluate those changes in MDD by collecting the data of clinical manifestations of MDD, the severity of symptoms, and response inhibition with emotional responding. Subjects with the non-affective disorder are supposed to show their ability of abstract thinking without abnormal emotional involvement even when they present the deficit of response inhibition after adding emotional element^20^. Some researchers proposed that a decrease in attention to olfactory stimuli in depression impaired olfactory identification and discrimination^21,22^, while the processing of the Stroop test is inseparable from attention. It was hypothesized that olfactory dysfunction was more likely to be associated with response inhibition than the mood in MDD.

## Experimental procedures

### Participants

We recruited 48 untreated patients with MDD and 33 healthy individuals matched on gender, age, level of education (see Table 1), and ethnicity (Han). The patients were assessed before treatment (baseline) and three months after the antidepressant treatment (follow-up), while the healthy individuals were also assessed twice.

**Table 1:** Comparison of the demographic information between MDD and HC groups.

	MDD	HC	*t*	*p value*
	*n* = *48*	*n* = *33*		
Gender			/	0.81^a^
Male	13 (27.1%)	10 (30.3%)		
Female	35(72.9%)	23(69.7%)		
Smoking habits			/	1.00^a^
Non-smoker	34 (70.8%)	24 (72.7%)		
Ex-smoker	14 (29.2%)	9 (27.3%)		
Age(y)	35.5 ± 11.8	33.9 ± 10.3	0.63	0.53
Education level(y)	11.9 ± 3.2	13.0 ± 3.6	1.41	0.16

MDD: major depressive disorder;. HC: healthy control;. mean (±SD) for the normal distribution data and number plus rate *(n,%)* for the qualitative data;. ^a^*p* was calculated by fisher’s exact test.

The patients were recruited from Shanghai Changning Mental Health Center or Shanghai Yangpu Mental Health Center, an inpatient psychiatric ward, which was registered in the period of 01/01/2017 to 07/31/2018. The diagnosis was confirmed by two psychiatrists independently, and the diagnosis of MDD with or without anxiety disorder was ascertained according to the International Statistical Classification of Diseases and Related Health Problems, 10th Revision (ICD-10)^23^. Inclusion criteria were: aged 18 to 60 years; Chinese Han ethnicity; the score of 17-item Hamilton Depression Rating Scale (HAMD-17) ≥ 17 points; sufficient socio-cultural background to understand the informed consent; first-diagnosed or long-term drug discontinuance (more than 12 weeks) defined as longer than five times of elimination half-life of any selective serotonin reuptake inhibitors (SSRIs)^24,25^; accepting one kind of SSRIs as the only intervention strategy. Exclusion criteria were: any other mental disorders in ICD-10; catching a cold within two weeks; history of chronic nasitis, nasosinusitis or nasal deformity; history of brain trauma, neurodegenerative disease or cerebrovascular disease; colour blindness or abnormal vision (uncorrected or corrected vision); pregnancy or postpartum period; drug abuse; alcohol consumption; current smoking; accepting modified electric convulsive treatment within 4 weeks; alternative treatment strategy employed in MDD patients who had no remission despite the adequate trial of SSRIs; pharmacological interaction between the current use of medications and antidepressants; suicide ideation (the score of the third item of HAMD-17 ≥ 3 points); olfaction experts, such as wine taster or perfumer.

The healthy individuals were recruited through advertisement. Inclusion criteria were: aged 18 to 60 years; Chinese Han ethnicity; the score of HAMD-17 < 7 points; the score of Hamilton Anxiety Rating Scale (HAMA) < 7 points. Exclusion criteria were: respiratory diseases; the history of mental disorder; color blindness or abnormal vision; pregnancy or postpartum period; drug abuse; alcohol consumption; current smoking; olfaction experts. Our study was based on approval by the Institutional Ethical Committee for clinical research of Shanghai Yangpu Mental Health Center (No. YJY2018–3), Shanghai, China. We ensured that all subjects were given an adequate understanding of the study, and written informed consent was provided according to the Declaration of Helsinki (1964). The study was approved by the Institutional Review Board at Shanghai Mental Health Center and the Institute of Psychology, Chinese Academy of Sciences.

Finally, thirty-five (72.9%) patients out of 48 remained in our cohort study after 3 months of antidepressant treatment. 13 (27.1%) patients dropped out. Meanwhile, 10 (20.8%) patients out of 48 had discontinued antidepressant medication (4 (8.3%) patients out of 10 worried about long-term side effects, 3 (6.3%) patients believed oneself to have been cured, and 3 (6.3%) patients were intolerant and had adverse drug reactions). Then, the rest 3 (6.3%) patients found it inconvenient to visit back in the hospital. 22 (66.7%) healthy individuals out of 33 completed the second round of assessment with 11(33.3%) healthy individuals being dropped out due to the inconvenience of visiting the hospital.

## Evaluation instruments

### Demographic and clinical information

The demographic and clinical information of eligible subjects was collected with a self-designed case report form including career, gender, age, education level, previous health status, current medication status, history of addiction (tobacco, alcohol, coffee, drugs).

The severity of symptoms was assessed by the HAMD-17^26^ and the HAMA^27^, two reliable and valid instruments.

### Chinese smell identification test

The Chinese Smell Identification Test (CSIT) was developed to evaluate the human olfactory function system. The CSIT had a test-retest reliability of 0.92 and was validated against the Sniffin’ Sticks Identification Test 16 and the University of Pennsylvania Smell Identification Test^28^.

The CSIT was tested in a quiet room with proper ventilation and without odor. Subjects were required to avoid eating garlic and drinking alcohol at breakfast, or exposing themselves in advance to strong smells in the garden, kitchen or other areas in the house. The process was arranged from 8:00 a.m. to 11:00 a.m. The Self-reported Olfactory Scale (SROS) was obtained before the CSIT using a 5-point scale from the worst to the best.

During the procedure of CSIT, participants were not allowed to eat food. After removing the cap of felt-tip pens which had been dipped into the different odorants respectively; they were placed 2 cm below subjects’ noses for 2 s. The ten pens which contained ten different kinds of odors; were placed in front of the subjects who should distinguish the proper smell from four choices of different odors. The interval time took 30 seconds. A correct odor choice was recorded as 1 point, while the wrong choice represented 0 point. For the consideration of avoiding smell fatigue, each individual had only one chance to complete the procedure. Total score ranged from 0 to 10, where a higher score represented a better olfactory function.

### Emotional stroop test

In the Emotional Stroop test (EST)^29^, the words were divided into three groups, containing neutral words (NEU), positive words (POS) and negative words (NEG) from scrutinized Chinese thesaurus^30^. Each group contained 20 words. Words in red or blue appeared randomly in the middle of the screen after a “+” symbol disappeared allowing an interval of 0.1 s for successive selection. The operation instruction was displayed on the screen at the very beginning. If the red or blue color appeared, the “F” or “J” key of the keyboard should be pressed respectively. Subjects were asked to place their left index finger on the “F” key and the right one on the “J” key. The E-prime 2.0 software was designed to define the blue color to be congruent with the negative words and the red to the positive words. If not, the selection would be programmed to be incongruent. The software recorded mean reaction time (MRT) and accuracy rate of the positive words and the negative words with word-color congruent (-C) or -incongruent (-I) as the POS-C, POS-I, NEG-C or NEG-I. The procedure was divided into three parts and forty trials in each part while one-minute intervals between each part. When comparing to healthy controls (HC), the MRT of each targeted variable indicated the different severity of response inhibition deficit and emotional response.

Considering the interaction of the MRT with the accuracy rate, Coomans F *et al*. suggest the parameter that governs the probability to answer fast is the parameter that governs the probability to answer correctly in their model. When performing fast processes in response to time-accuracy data, response time/accuracy is an alternative approach^31^. Therefore, we used the accuracy rate to correct the MRT. The adjusted MRT was represented as ΔMRT (MRT/accuracy rate). The procedure can better explain the correlation between response speed, rate of accuracy, and cognitive function. The longer the ΔMRT is, the worse the response inhibition will be.

## Statistical methods

IBM SPSS version 17.0 for Windows (Chicago Inc., USA) was performed for statistical analysis. All of the data were performed for normality and homogeneity of variance. Normal distribution data were represented as mean ± SD, then, the between-group variance was examined by Student’s t-test for two independent samples, while skewed distribution data was represented as median (IQR 25–75) performed by Mann-Whitney u Test. Fisher’s exact test was conducted to analyze the demographic data represented as a number plus rate (n,%). The significance level was defined as α = 0.05 (two-tailed). Potential statistical data correlations were conducted using multiple linear regression stepwise analysis within the CSIT, demographic information, HAMD-17, HAMA, and EST, the probability for stepwise through entry: 0.05 and removal: 0.10.

## Results

### The demographic information

Table 1 presents the descriptive statistics for the participant groups. There were no statistical differences in gender, smoking habits, age, or education level between the two groups (*p*’s > 0.05).

### The HAMD-17, HAMA, olfactory level of MDD before and after 3 months of treatment

The HAMD-17 and the HAMA of the MDD patients showed difference before and after 3 months of treatment (*p*’*s* < 0.05). MDD patients showed lower scores of the CSIT and SROS at baseline level (*p*’*s* < 0.05) compared to HC but no differences at follow-up level (*p*’*s* > 0.05) shown in Table 2.

**Table 2:** Comparison of the HAMD-17, HAMA, olfactory level between Baseline and Follow-up of MDD group. Mean (±SD) for the normal distribution data; median (IQR 25–75) for the skewed distribution data;. HAMD−17: the 17-item Hamilton Depression Rating Scale;. HAMA: the Hamilton Anxiety Rating Scale;. CSIT: the Chinese Smell Identification Test;. SROS: the Self-reported Olfactory Scale.

Scores	Baseline		*t/Z*	*p*	Follow-up		*t/Z*	*p*	MDD (baseline vs follow-up)		HC (baseline vs follow-up)	
	MDD *n* = 48	HC *n* = 33			MDD *n* = 35	HC *n* = 22			*t/Z*	*p*	*t/Z*	*p*
HAMD -17	20.9 ± 3.6	/	/	/	7.8 ± 4.1	/	/	/	15.4	<0.01	/	/
HAMA	18.7 ± 8.8	/	/	/	10.7 ± 4.3	/	/	/	5.51	<0.01	/	/
SROS	3 (3,4)	4 (3,5)	3.85	<0.01	4 (3,5)	4 (3,4)	1.28	0.20	4.10	<0.01	1.18	0.24
CSIT	7 (6,8)	9 (8,9)	4.09	<0.01	8 (8,9)	9 (9,10)	1.83	0.07	3.73	<0.01	1.19	0.24

### Comparison of the parameters of the EST between MDD and HC groups

**Figure 1:**
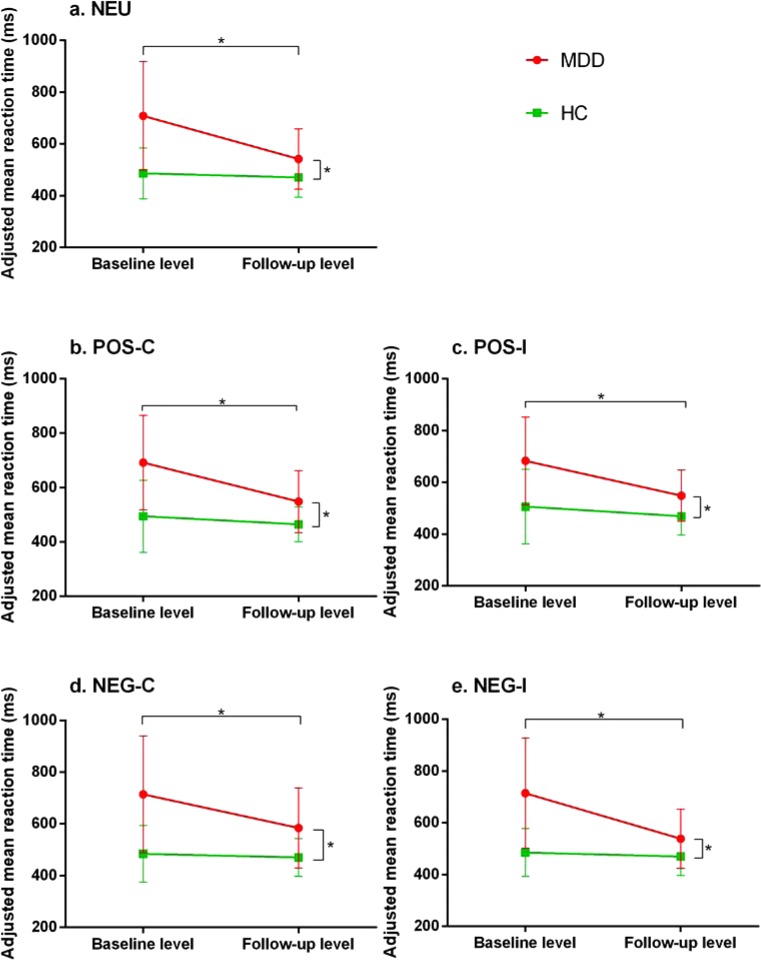
Difference between groups in the parameters of the emotional Stroop test (^*^P < 0.05). Adjusted mean reaction time= mean reaction timedisappeared allowing an interval ofaccuracy rate; NEU: neutral words; POS: positive words; NEG: negative words; -C: congruent; -I: incongruent.

MDD patients showed longer MRT and *Δ*MRT of all the parameters at baseline level compared to HC (*p*’s < 0.05), while the MRT and *Δ*MRT of MDD patients remained longer after the 3-month treatment compared to HC (*p*’s < 0.05). Shorter MRT and *Δ*MRT of the follow-up MDD patients were observed compared to the baseline MDD patients (*p*’s < 0.05). However, there were no difference of the MRT and *Δ*MRT in HC group between the first test and 3-month retest (*p*’s > 0.05). These results were shown in Fig. 1 and Table 3.

**Table 3:** Comparison of parameters of the EST between MDD and HC groups. MRT: mean reaction time; *Δ*MRT: adjusted MRT; NEU: neutral words; POS: positive words; NEG: negative words; -C: congruent; -I: incongruent.

	Baseline		*t/Z*	*p*	Follow-up		*t/Z*	*p*	MDD (baseline vs follow-up)		HC (baseline vs follow-up)	
	MDD *n* = 48	HC *n* = 33			MDD *n* = 35	HC *n* = 22			*t/Z*	*p*	*t/Z*	*p*
**NEU**												
MRT(ms)	690 ± 207	476 ± 102	6.16	<0.01	532 ± 114	460 ± 73	2.64	0.01	4.44	<0.01	0.64	0.52
Accuracy	0.97 ± 0.04	0.98 ± 0.02	0.59	0.56	0.98 ± 0.03	0.98 ± 0.02	0.75	0.46	1.25	0.22	0.05	0.96
ΔMRT(ms)	708 ± 210	486 ± 99	6.37	<0.01	542 ± 117	471 ± 77	2.77	<0.01	4.60	<0.01	0.62	0.54
**POS-C**												
MRT(ms)	670 ± 170	490 ± 132	5.14	<0.01	539 ± 109	455 ± 63	3.28	<0.01	4.02	<0.01	1.30	0.20
Accuracy	0.97 ± 0.06	0.99 ± 0.02	2.12	0.04	0.98 ± 0.03	0.98 ± 0.03	0.64	0.53	1.21	0.23	1.79	0.08
ΔMRT(ms)	692 ± 174	494 ± 132	5.52	<0.01	548 ± 114	465 ± 65	3.51	<0.01	4.27	<0.01	1.10	0.28
**POS-I**												
MRT(ms)	658 ± 162	495 ± 135	4.75	<0.01	538 ± 98	462 ± 71	3.16	<0.01	3.87	<0.01	1.05	0.30
Accuracy	0.97 ± 0.07	0.98 ± 0.03	1.37	0.12	0.98 ± 0.04	0.98 ± 0.02	0.02	0.83	1.29	0.20	0.52	0.60
ΔMRT(ms)	683 ± 170	506 ± 145	4.88	<0.01	549 ± 100	469 ± 73	3.46	<0.01	4.18	<0.01	1.25	0.22
**NEG-C**												
MRT(ms)	693 ± 217	475 ± 112	5.93	<0.01	573 ± 149	462 ± 77	3.70	<0.01	2.83	<0.01	0.48	0.63
Accuracy	0.97 ± 0.04	0.98 ± 0.03	0.73	0.47	0.98 ± 0.03	0.98 ± 0.05	0.11	0.91	1.34	0.19	0.30	0.77
ΔMRT(ms)	714 ± 226	485 ± 110	6.09	<0.01	584 ± 155	470 ± 73	3.74	<0.01	2.94	<0.01	0.54	0.59
**NEG-I**												
MRT(ms)	695 ± 209	475 ± 94	6.43	<0.01	531 ± 113	466 ± 75	2.40	0.02	4.62	<0.01	0.39	0.70
Accuracy	0.98 ± 0.04	0.98 ± 0.03	0.45	0.66	0.99 ± 0.03	0.99 ± 0.02	0.55	0.59	1.60	0.11	1.78	0.08
ΔMRT(ms)	714 ± 213	485 ± 93	6.61	<0.01	538 ± 115	469 ± 72	2.52	0.02	4.84	<0.01	0.65	0.52

MDD patients showed lower accuracy rate of POS-C at baseline level compared to HC (*p* < 0.05), while we observed no difference in the other four parameters (*p’s* > 0.05). There was no difference between MDD and HC at the follow-up level (*p’s* > 0.05). The accuracy rates of the observed MDD did not increase after the 3-month treatment (*p*’s > 0.05). See Table 3.

### Multiple linear regression stepwise analysis of the CSIT in the baseline MDD group

In this study, our aim was to associate the score of the CSIT with age (y), education level (y), the score of HAMD-17, HAMA, and SROS, and *Δ*MRT (ms) of the NEU, POS-C, POS-I, NEG-C and NEG-I before or after the 3-month treatment through the stepwise analysis of multiple linear regression. The regression model, which was finally constructed by age and the NEG-C (*F* = 11.89, *P* < 0.001, *R*^2^ = 0.346, adjusted *R*^2^ = 0.317) at baseline level, is shown in Table 4. The regression equation was finally obtained as follows:

$$y=10.676-0.063{x}_{1}-0.002{x}_{2},[{x}_{1}={\rm{the}}\,{\rm{age}}({\rm{y}}),{x}_{2}={\rm{the}}\,\varDelta \,{\rm{MRT}}\,{\rm{of}}\,{\rm{the}}\,{\rm{NEG}}-{\rm{C}}({\rm{ms}})]$$

**Table 4:** Results of multiple linear regression stepwise analysis of the CSIT in the baseline MDD group. B: non-standardized partial regression coefficient;. SE: Standard error;. Beta: standardized partial regression coefficient;. *R*^2^: multiple correlation coefficient-square;. *ΔR*^2^: adjusted *R*^2^.

	B	SE	Beta	*t*	*p*	*R*^2^	*ΔR*^2^
Constant	10.676	0.805		13.268	<0.001	0.346	0.317
Age(y)	−0.063	0.018	−0.447	3.555	0.001		
NEG-C (ms)	−0.002	0.001	−0.277	2.202	0.033		

## Discussion

We suggested that the total scores of the CSIT and SROS for olfactory function were discrepant between MDD and HC groups. Furthermore, the antidepressant treatment achieved the expected treatment goals by comparing the CSIT and HAMD-17 before and after 3-month treatment. The olfactory identification ability was recovered with the remission of MDD through adequate dosage and duration of antidepressant treatment^32^. Until today, olfactory evaluation has always been neglected by the physicians during diagnosis course in China. The CSIT is specially designed for the olfactory characteristics of the Chinese population, compared with the University of Pennsylvania Smell Identification Test (UPSIT) and the Sniffin’Sticks Identification Test 16 (SS-16) commonly used in the world, it has a good reliability^28^. The CSIT made up for the shortage of olfactory evaluation based on the Chinese population.

Some experts suggested that the subjects were just the patients with MDD in previous studies, and then reached the conclusion that the lower odor identification performance belongs to these individuals with MDD^33,34^. However, mild or moderate depression do not accompany with olfactory dysfunction^7^. We doubted that the severity of symptom might be related with olfactory processing even though diagnosed as MDD. Therefore, we studied the relationship between the CSIT and the HAMD-17 in untreated MDD through multiple linear regression stepwise analysis. As a result, the correlation between the scale and the olfactory function did not reach consistency as expected. The reason for this is that the severity of depressive symptoms might not affect olfactory function, course, and duration of depression^35^.

The neurobiology may explain the olfactory dysfunction of MDD. The pathological basis of emotional regulation, olfactory function, and response inhibition share common functional regions such as the OFC and ACC^36–38^. Studies indicate that pathological changes in depressive state are associated with a large number of neurobiological changes leading to the dysfunction, which point to those interactive functional regions^39^. Those neurobiological changes strengthen the connections of brain regions with depression while weakening connections between the limbic system and neural networks exposed to the stress in the prefrontal cortex^40^.

To cope with the endless flow of information from sensory perception, the brain has to develop a simple and effective processing program within limited neuronal connections, which is the so-called “selective attention”. Once a sensory input is defined as a non-correlated signal, the response inhibition reduces the expression of a distractor, and then prevents it from reaching the response system in order to reduce unnecessary interference. Therefore, interference suppression becomes the core concept in the response inhibition, which is called inhibition of competing distractors or inhibition of competing for automatic response^41^. Emotional setting and shifting involved in Stroop test can solve the assessment of competing for automatic response simply (such as the central dimension of the EST)^42^. In the EST, the POS-C, POC-I, NEG-C and NEG-I words reflect response inhibition and emotional response under different color-emotional word interference, while the NEU words reflect responding baseline only. Our results showed that even with the remission of the disease, the NEU, POS-C, POC-I, NEG-C and NEG-I of MDD patients ameliorate after the treatment but remain worse than those of control participants. However, we cannot distinguish accurately whether the improvements are caused by each emotional competing distractor. Then, we performed a regression model showing that the NEG-C was associated with the CSIT at the baseline level. Olfactory deficiency is more likely to be affected by negative emotional responding, but the deficit of negative emotional responding cannot be recognized by the HAMD-17 in this model. Our results may be explained through two systematic reviews of the EST: emotional Stroop effect seems to rely more on a slow disengagement process than on a fast^43^ while robust depression-related Stroop effects play an imperative role in negative stimuli for clinically depressed versus healthy people^44^.

These pathological and physiological changes not only interfere with the processing of the emotional and cognitive functions in depression, but have an inseparable impact on the olfactory system as well^45^. This statement was also supported by our study. We observed that the MDD patients, not only had mood dysregulation, but also their response inhibition and olfactory functions, were significantly impaired. The emotional Stroop effect reflects the process of emotional responding and the response inhibition^29^, however, little evidence was shown on neuropsychological interaction of the olfactory function in MDD simultaneously. In the olfactory correlation analysis, the response inhibition deficit with emotional responding was negatively correlated with the impaired olfaction, especially in the negative emotional responding, as well as age. It is consistent with the current findings that age is a high-risk factor for olfactory dysfunction in depression^32^. Our findings are inconsistent with the severity of depression measured by the HAMD for predicting olfactory function^46^, on the other hand, the severity of olfactory impairment is related to the*Δ*MRT of NEG-C in the EST with moderate correlation. Another outcome revealed that the response inhibition could not return to the average level after the 3-month treatment. The cognitive impairments in MDD are trait-like phenomena^47^, which was duplicated by our study.

Although similar questions have been addressed before, it is still necessary to establish data on original olfactory test tools targeting Chinese people to conduct further olfactory-related research on Chinese. Meanwhile, there has been no association of such olfactory research on depression with cognitive function in China. However, our study has many limitations. First of all, the sample size of this study was relatively modest, which does limit the power of the test, so we need to expand the sample size in the future for this series of research. Other than that, for a more detailed discussion on the olfactory function in depression, more influenced factors can be taken into consideration, such as gender, the severity of depression, course and duration of depression. Moreover, though the follow-up subjects had clear curative effects, patients with refractory depression or poor treatment compliance^48^ hardly can be included in real-world study even when they do meet the inclusion and exclusion criteria, which needs to be considered in the high-quality design.

We indicate olfactory identification deficit in the patients with MDD. However, Swiecicki L *et al*.^49^ did not find that difference between the HC and recurrent depressive disorder. It is suggested that acute episode of the disease may have a different phenotype of chemical sense, for the HAMD-17 (20.9 ± 3.6) was higher in our study compared to their description of the severity of MDD symptom using the HAMD-21 (15.2 ± 1.6).

The reduced volume of olfactory bulb of depressive patients was observed from the perspective of organic brain syndrome^50–52^. A previous study divided patients with depression into two subgroups–therapy responders and nonresponders, and found the therapy responders exhibited no significant difference in olfactory bulb volume compared to HCs. However, that of nonresponders was 23% smaller compared to responders^50^. They suggested that odor identification is significantly impaired in depressive episode and recovered from treatment. We similarly excluded the MDD patients who had no remission despite the adequate trial of SSRIs (nonresponders). Therefore, combined with their findings, we assume that the olfactory bulb structure may not, or just temporarily, be influenced and functionally normalized to HC level at follow-up.

Another study found that structural markers, such as the olfactory bulb volume may relate to the vulnerability to depression while functional markers reflect current symptomatology^52^. This hypothesis deserves to be verified by monitoring each of them at different times, including threshold, identification and discrimination while functional imaging of olfactory bulb volume was employed.

In conclusion, patients with MDD showed decreased olfactory ability, but this phenomenon only occurred in the acute phase of MDD. Correlation analysis indicated that the MRT of negative stimuli and age may be two biological predictors of olfactory perception prognosis in patients with MDD. With the remission of the disease, olfactory dysfunction returned to be normalized. The present study suggests that the remission of MDD may be regarded as a response phenotype of brain function, including the cognitive function and olfactory function but not the emotional responding. The causes of drop-outs in MDD group were mainly due to drug discontinuation. This is also close to the drop-out rate of treatment in the real-world treatment process^53^.
